# Does accreditation improve pro re nata benzodiazepines administration in psychiatric inpatients? Pre-post accreditation medical record comparison

**DOI:** 10.1186/s13033-017-0124-8

**Published:** 2017-02-02

**Authors:** Mohammed Abdullah Al-Sughayir

**Affiliations:** Psychiatry Department, College of Medicine, King Saud University, PO Box 21525, Riyadh, 11485 Saudi Arabia

**Keywords:** Accreditation, Benzodiazepines, Inpatient, Mental health, Pro be nata, Quality, Psychiatry, Saudi Arabia

## Abstract

**Background:**

In psychiatric inpatients, administration of pro re nata benzodiazepines is a common practice. Benzodiazepine use is associated with potential complications of risk of abuse, cognitive impairment, and falls. An interest in accreditation is growing rapidly among many countries to enhance the quality of health care services. We aimed to investigate whether hospital accreditation drives improvements for administered pro re nata benzodiazepines in psychiatric inpatients.

**Methods:**

The study reviewed medical records of consecutive hospital admissions for pre- and post-accreditation comparisons of PRN benzodiazepine medications in two acute mental health wards at a teaching general hospital. Data obtained from the 12-month-post-accreditation period (July 2011–June 2012) were compared with those from the 12-month-pre-accreditation period (July 2009–June 2010). The adoption of accreditation standards occurred over a 12-month period in the middle of the study (July 2010–June 2011). Compiled information included demographics, diagnosis, assessment, and LOS. All identified charts were reviewed; there were no exclusion criteria. Patients were not contacted.

**Results:**

There was a statistically significant (P < 0.002) reduction of approximately 22% in the number of administered PRN benzodiazepines. Post-accreditation, the average number of PRN benzodiazepines administrations per patient, was 4.83 ± 2.1 compared to 6.19 ± 3.4 pre-accreditation. There was no significant difference between the two genders. The highest average quantity of PRN benzodiazepines administered was during the time interval of 18–24 h.

**Conclusion:**

Accreditation may have a positive impact on the process of administering PRN benzodiazepine medications in psychiatric inpatients.

## Background

Benzodiazepines (BDZs) were developed in 1962 as a treatment for anxiety symptoms. Because of rapid tranquilizing effect, BDZs are commonly used for the control of agitation, disruptive behavior, and insomnia in psychiatric inpatients [1]. More recently there has been a shift away from using the sedative side-effects of antipsychotics to using benzodiazepines that sedate with minimal side-effect risks [2]. Benzodiazepines such as lorazepam are recommended as the drugs of first choice when rapid tranquillization is required because of their sedative effects [3]. However, benzodiazepine use is also associated with potential complications of tolerance, dependence and withdrawal symptoms, risk of abuse and cognitive impairment [2]. Dependence can develop to therapeutic doses of benzodiazepines, after 4–6 weeks of regular usage, but it may develop more rapidly to very high doses administered for a shorter period of time or in the case of individuals who have been previously dependent on other sedatives or alcohol [4]. Despite guideline precautions, long‐term prescription of BZDs is still a common treatment pattern [5, 6]. Clinicians’ over prescriptions of BZDs may result in abuse problems [7].

The present study focused on BDZ medications because their clinical relevance to psychiatric inpatients. There are many adverse effects of BDZs on psychiatric inpatients including cognitive impairment, delirium, psychomotor slowing, risk of respiratory distress, oversedation, falls and hip fractures [8, 9].

Interest in accreditation of health care organization (HCOs) is growing rapidly among developing countries [10]. Saudi Arabia was one of the first countries in the eastern Mediterranean region to implement health care accreditation standards [11]. Several governmental hospitals in Saudi Arabia have received accreditation from different international accreditation bodies [12]. King Khalid University Hospital (KKUH) in Riyadh, the site of the study, obtained two cycles of accreditation from Accreditation Canada International (ACI). The first was in February 2011, and the second was in May 2014. The ACI is a non-governmental quality organization that offers health care improvement services worldwide.

In psychiatric inpatient services, administration of pro re nata (PRN, or ‘as required’) psychotropic medication is a common practice. The rate of administered PRN medications to patients in psychiatric units in the United States of America is 35%, Canada 50%, Britain 50% and Australia 80% [13, 14]. On admission, about 75% of mental health patients were routinely prescribed PRN medications [15]. Unnecessary reliance on PRN psychotropic medications for psychiatric inpatients can undermine the quality of care. To ensure efficient care in inpatient psychiatric services, standardized practice guidelines for PRN medications are essential. The clinical practice guidelines for PRN medications eliminate differences in implementation and reduce a medication-related morbidity, which is strongly associated with PRN medications.

Pro re nata BDZ medications is important and under-researched clinical intervention used in psychiatric wards. Little is known about the effect of the accreditation process on PRN benzodiazepine medications in psychiatric inpatients. Prescribing PRN benzodiazepine medications in psychiatric inpatients in Saudi Arabia have never been reported [16, 17]. Thus, this study attempted to investigate whether hospital accreditation drives improvement of administering PRN benzodiazepine medications for psychiatric inpatient care.

## Methods

### Design

The study reviewed medical records of consecutive hospital admissions for pre- and post-accreditation comparisons of PRN benzodiazepine medications in two acute mental health wards.

### Site

The inpatient psychiatric units at King Khalid University Hospital (KKUH) in Riyadh, Saudi Arabia, which is the only public teaching hospital in Riyadh City with an 800-bed capacity. The psychiatric units comprise 22 mental health beds (11 for each sex) in locked-door wards, and adequately staffed with psychologists and social workers along with medical and nursing personnel. Patient admissions are usually through the emergency department, outpatient clinics, and rarely from medical wards. Hospitalization includes stabilization of crisis presentations, planned diagnostic assessments, and brief intensive treatment.

### Subjects

Psychiatric inpatients admitted during the 12-month-post-accreditation period (July 2011–June 2012) were compared with those from the 12-month-pre-accreditation period (July 2009–June 2010). All identified charts were eligible for review including patients leaving against medical advice; there were no exclusion criteria. However, to maintain independence of observations, only the first hospitalization per patient during the study period was included and the charts of patients who were readmitted during the study period (three patients in the pre-accreditation and two patients in the post-accreditation periods) were excluded. Patients were not contacted.

## Data collection

A structured data collection sheet has been made including the following factors for each patient demographics, diagnosis, assessment, and administered PRN benzodiazepine medications. The psychiatric inpatient case register in each unit was accessed to identify all administered PRN benzodiazepine medications to in-patients admitted during the two study periods. Data in the case register are recorded by nursing staff under the direct supervision of an expert head nurse. After patient’s discharge, the medical file is forwarded to the Medical Record Department at KKUH. Clinical data were extracted in October 2012 by the author from patients’ files at the Medical Record Department. Data were paper record-based information. The quality of data records was identified and assessed based on the availability and legibility of a detailed documentation for all admissions in both study periods. The psychiatric nurses, under the supervision of the unit head nurse, recorded the required data in legible English. The data were then coded and entered into statistical software.

### Intervention

The accreditation process, which is a system of strategic planning to promote the quality of the clinical practice. The accreditation program included 18 mental health standards focusing on patients’ safety, a recommendation to adopt clinical practice guidelines for PRN medications (Table 1), biopsychosocial multidisciplinary team (MDT) approach with an objective assessment of symptoms severity through the Brief Psychiatric Rating Scale (BPRS), rapid evaluation, and clear discharge plan. The standards incorporated the Plan-Do-Check-Act circle. The adoption of accreditation program occurred over a 12-month period (July 2010–June 2011) in the middle of the study. A team of surveyors examined hospital’s compliance with the Accreditation Canada International standards during an onsite survey. Hospital performance was assessed based on reviewing guidelines, interviewing staff, and conducting tracers. Based on these findings, the hospital as a whole was awarded accreditation in February 2011.

**Table 1:** The clinical practise for the guidelines for the administration of PRN psychotropic applied at KKUH psychiatric in patients^a^

After patient admission, all current medications should be documented and reciewed by the admitting team for medication reconciliation			
Use of regular medications for individual patients as PRN is always recommended. Polypharmacy is discouraged			
When handling a patient’s difficult behaviour, before resorting to PRN medications, alternative interventions (e.g., counseling) should be attempted			
For each patient, the treating psychiatrist should complete the medication orders with the required regimen of PRN medication as soon as possible			
Patient accepting oral PRN medications and appropriately responding to it should not be given an injection			
Administered PRN medication and its response should be clearly documented			
After administered PRN medications, the nurse in charge should monitor the vital signs at least hourly and watch for extrapyramidal side effects			
If the nurse has any concern, he/she should immediately inform the treating phychiatrist and ask for a medical evaluation			

^a^With permission from Phychiatry Department, College of Medicine, KSU

### Analysis

The collected data were entered into a spreadsheet for analysis. Statistical analysis was conducted using Statistical Package for the Social Science (SPSS) version 15 software for Windows (SPSS Inc., Chicago, IL, USA). The Mann–Whitney test was used to compare the means from 2 independent groups. A P value of <0.05 indicated statistical significance.

## Results

There were 182 patients, during the post-accreditation period, compared to 177 patients during the pre-accreditation period. Table 2 shows the socio-demographic and clinical characteristics of the study populations for the two study periods. There were no statistically significant differences (*P* > 0.05; for all comparisons).

**Table 2:** Comparison between the demographic and clinical characteristics of pre- and post-accreditation patients

Variable	Study period		P value^a^
	Pre-accreditation n = 177 (%)	Post-accreditation n = 182 (%)	
Sex			
Male	82 (46.3)	84 (46.2)	0.999
Female	95 (53.7)	98 (53.8)	
Age (years)			
<25	50 (28.2)	59 (32.4)	0.590
25–50	104 (58.8)	104 (57.1)	
>50	23 (13.0)	19 (10.5)	
Marital status			
Single	98 (55.4)	112 (61.5)	0.352
Divorced/separated	20 (11.3)	22 (12.1)	
Married	59 (33.3)	48 (26.4)	
Diagnosis			
Organic mental disorders	6 (3.4)	11 (6.0)	0.089
Non-affective psychosis	78 (44.1)	61 (33.5)	
Affective psychosis	73 (41.2)	94 (51.6)	
Others disorders	20 (11.3)	16 (8.8)	

^a^Level of statistical significance is 5%

Post-accreditation, the average number of PRN benzodiazepines administrations per patient, was 4.83 ± 2.1 compared to 6.19 ± 3.4 pre-accreditation. There was a statistically significant (P < 0.002) reduction of approximately 22% in the number of administered PRN benzodiazepines. There was no significant difference between the two genders.

The average quantity of PRN benzodiazepines administered in each 6-h interval during the day, throughout the whole period of admission, was also investigated for the two study periods. The highest average quantity of PRN benzodiazepines administered was during the time interval of 18–24 h (Table 3).

**Table 3:** Comparisons of the average number of tablets of PRN benzodiazepines administered every 6-h intervals throughout the day

Timing (24 h)	Study period		P value
	Pre-accreditation	Post-accreditation	
000–0600	2.41 ± 1.2 (70)	1.85 ± 1.0 (66)	0.003^a^
0600–1200	None	1.44 ± 0.5 (16)	N. A.
1200–1800	2.0 ± 0.8 (4)	1.60 ± 0.6 (50)	0.263
1800–2400	5.05 ± 2.4 (128)	3.30 ± 1.4 (152)	<0.001^a^

^a^Statistically significant at 5% level of significance

In both study periods, lorazepam was administered most often followed by diazepam.

The most common reported reasons for administration of PRN benzodiazepines in both study periods were agitation, insomnia, and aggression.

## Discussion

Accreditation of health care organizations encourages achieving and validating a high quality of patients’ care. The present study focused on the actually administered rather than on the prescription pattern of PRN benzodiazepines.

The socio-demographic and clinical characteristics of the study populations for the two study periods appear to have no confounding effects in the present study, as there were no statistically significant differences for all comparisons. However, research has shown that psychiatric diagnosis has a limited influence on PRN administration. The main factors that affect the use of PRN medications in psychiatric inpatients are the severity of behavioral disturbances, the availability of alternative interventions, the ward environment, and nursing staff characteristics rather than the diagnosis [18].

There was no change in the number of nursing staff at KKUH psychiatric wards in the two study periods.

The reported means of PRN medications in psychiatric inpatients in the international studies were 10–12 administrations per patient [19, 20]. In the present study, the average number of PRN benzodiazepines administrations per patient post-accreditation was 4.83 ± 2.1 compared to 6.19 ± 3.4 PRN benzodiazepines administrations per patient before accreditation. There is a reassuring significant reduction of approximately 22% in the number of administered PRN benzodiazepines per patient. This reduction can be explained by many collaborating factors. First, the positive effect of the clinical practice guidelines for the administration of PRN psychotropic medications at KKUH psychiatric inpatient units. Second, the role of the biopsychosocial multidisciplinary management approach. Third, the participation of a clinical pharmacist in the regular review of patients’ treatment to ensure appropriateness of ongoing prescribed medication regimens and in the education of nursing staff regarding medication choice and alternative treatment options. Fourth, nurses perceived the accreditation and the clinical practice guidelines favorably. Before resorting to PRN medications, nurses were encouraged to utilize a positive nurse-patient relationship and to apply non-pharmacological alternative interventions like face-to-face de-escalation and supportive contacts. However, the content of such interventions is difficult to validate. Further investigation is required to explore the successful factors embedded in the alternative interventions.

It would have been better if the study had investigated the rates of seclusion and restraints to know whether there had been an accompanying increase in the alternative methods of inpatients containment. However, some researchers found that while the rate of administered PRN medications decreased significantly, there was no accompanying increase in the other methods of inpatients containment; seclusion and restraint [21].

In concordance with previous studies [19, 22], of the PRN benzodiazepines in this study, lorazepam was administered most often in both study periods.

The most common reported reasons for the administration of PRN benzodiazepines in this study were agitation, insomnia, and aggression. This is consistent with previous studies [23, 24].

The average quantity of PRN benzodiazepines administered per patient between 18:00 and 24:00 h was five in the pre-accreditation period, and it was reduced to 3.3 during the post-accreditation period. In accordance with previous studies [24, 25], the most common time for a PRN benzodiazepine medication to be administered was between 18:00 and 24:00 h. This time of the day is consistent with settling patients at bedtime because sleep disturbances are often present in acute mental illness. This temporal pattern of PRN sedation allows an opportunity for initiating behavioral interventions to reduce the need for unnecessary PRN benzodiazepines.

The study has certain limitations that should be considered. It represents data for only one center with an inherent selection bias. It is a record-based retrospective study with questions on the reliability of documentation. However, the initial information of this study can provide a view regarding accreditation effect on medical practice in KSA, which may assist in the future planning of psychiatric services in the country. In KSA, national standard practice guidelines to the use of PRN medications in mental health services should be established and implemented.

To support the findings of this study, multicenter research with a larger sample size is substantially needed. Future studies need to focus on the impact of quality measures on prescriber characteristics, whether BDZs were discontinued before discharge and the duration of BDZs after discharge.

In conclusion, findings indicate that hospital accreditation may have a positive impact on the process of administering PRN benzodiazepine medications in psychiatric inpatients.

## Abbreviations

PRN: Pro Re Nata (as needed)
ACI: accreditation Canada international
SPSS: Statistical Package for the Social Science
SD: standard deviation
BPRS: brief psychiatric rating scale
KKUH: King Khalid University Hospital
KSU: King Saud University
MDT: multi-disciplinary team
